# Microbial profiling of black soldier fly larvae reared on substrates supplemented with different mineral sources originating from phosphorus recycling technologies

**DOI:** 10.1186/s42523-025-00380-5

**Published:** 2025-02-11

**Authors:** Henry Reyer, Manfred Mielenz, Gürbüz Daş, Cornelia C. Metges, Klaus Wimmers

**Affiliations:** 1 Research Institute for Farm Animal Biology (FBN), Wilhelm-Stahl-Allee 2, 18196 Dummerstorf, Germany; 2 Faculty of Agriculture and Environmental Sciences, Professorship of Animal Breeding and Genetics, University of Rostock, Justus-Von-Liebig-Weg 6, 18059 Rostock, Germany

**Keywords:** *Hermetia illucens*, Sewage sludge, Insect production, Recyclates, Biochar

## Abstract

**Background:**

Innovations to establish agricultural value chains utilising side streams and their reintegration into the feed and food supply are of great importance. Recyclates derived from biomass and waste are therefore becoming increasingly important as sources of nutrients. The larvae of the black soldier fly (BSF; *Hermetia illucens*) demonstrate considerable potential as livestock feed due to their ability to utilise a wide range of organic substrates. In this study, BSF larvae (BSFL) were reared on four different substrates: chicken feed diet (CD), high-fibre Gainesville fly diet (FD), or FD supplemented either with biochar (FD + BCH) or single superphosphate (FD + SSP) recyclates from sewage sludge processing. To validate the hypothesis that endogenous and substrate-associated microbiota significantly contribute to substrate conversion, the microbiota profiles of BSFL gut and frass were analysed by *16S* rRNA gene amplicon sequencing. Results were associated to the different substrates as well as body composition, growth performance data, and mineral concentration of the larvae.

**Results:**

The CD substrate was superior in terms of larval growth, although it caused a lower microbial alpha diversity in the larval intestine and frass compared to FD, with a dominance of *Morganellaceae* and families of *Lactobacillales*. The addition of the two sewage sludge derived products to the FD substrate significantly increased the calcium content of BSFL, while the phosphorus content was only increased by the addition of SSP. The shifts in the microbiota profiles of BSFL gut and frass indicated that BCH contributed to the regulation of the microbial milieu with suppressing the growth of potentially pathogenic microbes. The addition of SSP resulted in an enrichment of microorganisms with attributed phosphate-solubilising properties such as *Pseudomonas* and fungal species, likely being responsible for improving the bioavailability of phosphorus from the substrate.

**Conclusions:**

The results demonstrate the high adaptability of the BSFL and its ability to change the substrate through specific microbiota in such a way that conditions are created for an optimal nutrient supply and thus growth of the larvae.

**Supplementary Information:**

The online version contains supplementary material available at 10.1186/s42523-025-00380-5.

## Background

Sewage sludge–derived products are a rich source of minerals such as phosphorus (P) and calcium (Ca), and the corresponding products are considered valuable fertilizers [1, 2]. Technological approaches have emerged to produce substrates from sewage sludge with improved product safety, e.g. reduction of heavy metals and pathogens that allow the establishment of nutrient cycles [3]. In the pyrolysis process, biochar is produced by heating the sewage sludge at temperatures between 300 and 700 °C. The sewage sludge ash can be used to produce mineral rich single superphosphate (SSP). The physicochemical properties, nutrient, and heavy metal contents of recycled sewage sludge are strongly influenced by the processing conditions [3, 4]. Recent studies have emphasized the efficacy of insect bioconversion in the management of sewage waste and the subsequent utilizations of its derivatives to establish nutrient cycles [5, 6]. In particular, the black soldier fly larvae (BSFL, *Hermetia illucens*) has recently received considerable attention due to their ability to convert various substrates into larval proteins, lipids, mineral-rich biomass and other valuable materials [7, 8]. However, since BSF are considered farm animals, their feedstuff is subject to European feed regulations laid down in Regulations (EC) No 1069/2009 and (EU) No 142/2011. Consequently, the use of manure or sewage sludge products as feed ingredients for insects used for feed and food are prohibited. Nevertheless, due to the protein and lipid-rich body mass of BSFL generated with high conversion efficiency, the use of larvae is considered a novel feed source for a variety of livestock species [9, 10].

On average, about 40% and 30% of the BSFL dry matter are crude proteins and fats, respectively [11]. The nutritional profile of the larvae depends on the stage of development and the downstream processing technique, but mainly on the composition of their feeding substrate. The selection of the substrates for BSFL production can reduce competition with human food sources and influence sustainability [12]. In this regard, the use of organic waste streams and by-products, especially those rich in fibres, is beneficial and can additionally improve the substrate texture and structure necessary for larval development and optimise the efficiency of bioconversion [13]. The substrate composition also influences the activity of various digestive enzymes and the expression of a repertoire of antimicrobial peptides in the larval gut [14, 15]. In addition to the intrinsic mechanisms of the larvae, the gut microbiota of BSFL, which includes bacteria, archaea and fungi, contributes to the decomposition of biomass and promotes their conversion into nutrients that can be absorbed by BSFL [16]. The microbial community and its activity are therefore strongly determined by the initial substrate, but the larvae themselves also influence the composition and colonization of the substrate with bacteria and fungi [17, 18]. It has been shown that sterilized BSFL did not grow on autoclaved diets suggesting that microbiota in the substrate and larva-associated microbes synthesize nutrients, which are essential for larval growth [19]. Significant contributions to degradation of biomass have so far been described for species of the genera *Actinomyces*, *Dysgonomonas*, and *Enterococcus*, as well as for representatives of *Morganella* and *Enterobacteriaceae* [16]. Another functional contribution of the gut microbiota is the maintenance of gut health and protection against pathogens, which includes the elimination of harmful microbes and the promotion of beneficial microbial colonization. Appropriate microbial strategies to combat pathogens include the production of antimicrobial substances such as bacteriocins and microcins and of metabolites such as short-chain fatty acids and lipopeptides. Rearing of BSFL on pig slurry revealed several taxa, including *Oblitimonas*, *Terrisporobacter*, *Paenalcaligenes*, *Pseudomonas*, *Savagea*, and *Sphingobacterium*, which potentially antagonise *Staphylococcus aureus* and *Salmonella spp* colonization [20]. For fungi, growing BSFL on compost has been shown to result in the complete elimination of mycelial fungi, including moulds, from the substrate and helps to create a more favourable fungal environment [17]. Depending on the substrate, mainly representatives of the fungal genera *Pichia*, *Cyberlindnera*, *Saccharomycodes*, *Yamadazyma*, *Saccharomyces* and *Scopulariopsis* were identified, with *Pichia kudriavzevi* being the dominant species [21].

This study builds on the BSFL trial by Seyedalmoosavi et al. [7], in which growth performance and mineral accumulation were characterized by the supplementation of sewage sludge-derived products to the BSFL feeding substrates. The aim of the present work was to investigate the effects of dietary fibre and the addition of Biochar and SSP with different mineral profiles on the intestinal and frass microbiota of BSFL. The experimental substrate groups were a modified fibre-rich Gainesville fly diet (FD), and FD supplemented with either biochar (FD + BCH) or single superphosphate (FD + SSP) compared to a commercial broiler diet (chicken feed diet, CD). Consequently, the hypothesis of the study was that the microbial diversity and the abundance of specific taxa were higher in both larvae and frass when reared on FD substrates compared to CD substrates. Furthermore, it was hypothesized that the microbial contribution to substrate conversion, including the abundance of fungi, can be enhanced by supplementing the substrate with certain minerals derived from recycled sewage sludge.

## Results

The BSFL reared on CD substrate had a significantly higher body mass and a higher dry matter (DM) content compared to those grown on the FD-based substrates (Table 1).

**Table 1:** Body mass and concentrations of minerals in black soldier fly larvae reared on chicken feed diet (CD), fly diet (FD), and FD supplemented with 4% biochar (FD + BCH) or 3.6% single-superphosphate recylates (FD + SSP)^1^

Larval phenotype	CD	FD*	FD + BCH*	FD + SSP*	SE	*P*-value
Body mass (mg/larva)	231.9^a^	103.2^b^	99.5^b^	89.7^b^	4.05	0.001
Dry matter (DM; %)	36.3^a^	28.6^b^	29.3^b^	27.9^b^	0.62	0.001
Calcium (g/kg DM)	32.02^d^	45.40^c^	53.24^b^	64.59^a^	1.42	0.001
Phosphorus (g/kg DM)	6.66^b^	7.09^b^	7.24^b^	10.89^a^	0.35	0.001
Magnesium (g/kg DM)	2.75^c^	3.29^b^	3.63^ba^	3.96^a^	0.11	0.001
Sodium (g/kg DM)	0.74^c^	0.94^b^	0.94^b^	1.27^a^	0.03	0.001
Potassium (g/kg DM)	8.98^b^	10.83^b^	11.01^b^	14.46^a^	0.66	0.001
Iron (mg/kg DM)	263.7^b^	144.6^b^	556.6^a^	339.2^ab^	64.0	0.002
Zinc (mg/kg DM)	127.5^a^	95.6^b^	107.4^b^	98.5^b^	3.45	0.001
Copper (mg/kg DM)	11.78	9.94	12.22	10.14	0.616	0.037
Cadmium (mg/kg DM)	0.183^c^	0.628^b^	0.771^a^	0.737^ab^	0.033	0.001
Lead (mg/kg DM)	0.175^c^	0.225^c^	1.160^a^	0.536^b^	0.049	0.001
Manganese (mg/kg DM)	388.9^a^	282.4^b^	303.6^b^	302.8^b^	10.1	0.001

^1^The statistical analyses comprised six replicates (boxes) per substrate. Statistical analysis of data was performed using analysis of variance with diet and block effects.

*Data shown are partly based on Table 2 in Seyedalmoosavi et al. [7]

^abc^Different superscripts within a row indicate significant differences in the parameter between the substrates (*P* < 0.05).

P-values shown in the table represent the diet effects.

No significant differences in growth traits were observed between the larvae reared on FD substrates with and without supplementation of different sources of recycled sewage sludge. The larvae rearing on the different substrates revealed differences in their mineral profile. When reared on CD, the larvae exhibited the lowest concentrations of Ca, Mg, and Na compared to all other groups. The concentrations of Ca, P, Na, and K were highest in the larval body of the FD + SSP group compared to the other substrates. In contrast, the iron content of the larvae was numerically highest in FD + BCH, while the zinc concentration in all larvae reared on FD substrates was lower than in the CD group.

**Fig. 1:**
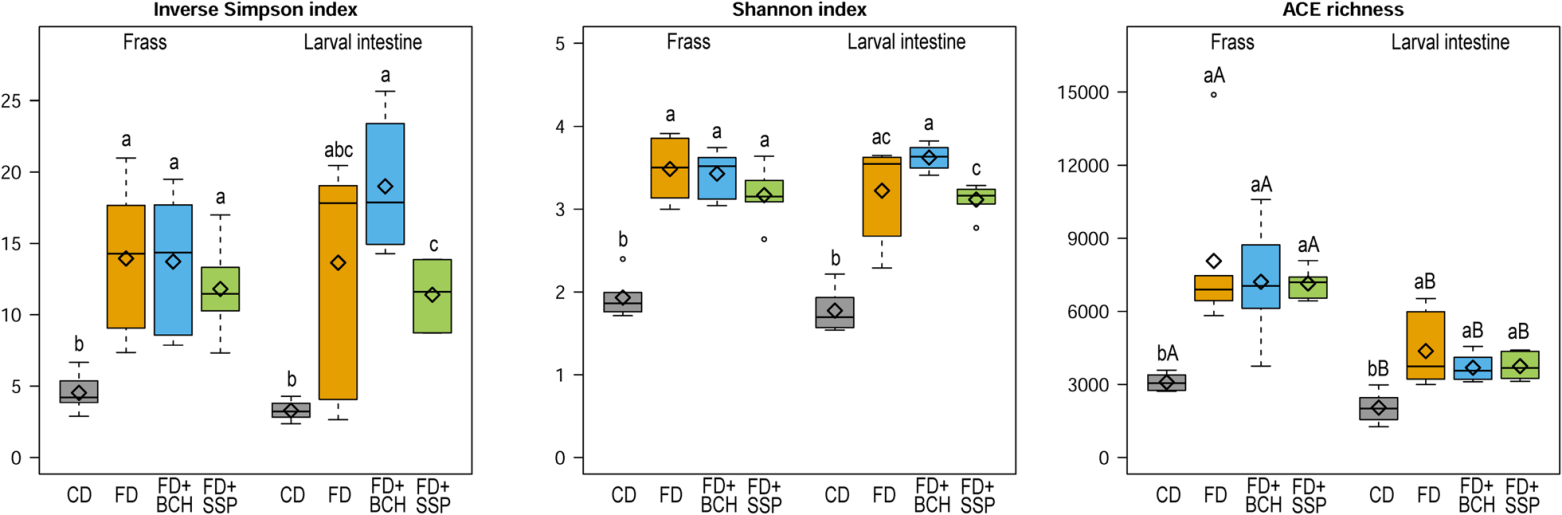
Alpha diversity indices of frass and larval intestine microbiota profiles across different feeding substrates including chicken feed diet (CD), fly diet (FD) and FD supplemented with biochar (FD + BCH) or single superphosphate (FD + SSP) recyclates. The boxes give the 25th to 75th percentiles, the line the median, the diamond the mean, the whiskers the minimum and maximum values and the circles the outliers. ^abc^ and ^ABC^ indicate significant differences (*P* < 0.05) between feeding substrates within frass or larvae and for the same feeding substrate between frass and larvae, respectively

Larval intestine and frass samples were collected from 18–20 day old BSFL reared on the different substrates to characterize the endogenous and substrate-associated microbiota. To establish the baseline microbiota composition, one sample of the initial substrate after a 24-h pre-soaking treatment was also analyzed. Microbial community composition was assessed through targeted sequencing of the *16S* rRNA gene hypervariable region 4. Microbial alpha diversity indices, including species richness (abundance-based coverage estimator; ACE) as well as species diversity and species evenness (reflected by Inverse Simpson index and Shannon index), were assessed in the frass and intestine of larvae reared on different substrates (Fig. 1). Alpha diversity indices were lower for CD compared to FD-based feeding substrates in both frass and larval samples, with the exception of the inverse Simpson index in the larval intestine (CD vs. FD). However, further differences were observed in the larval intestine but not in the frass samples between FD supplemented with BCH or SSP. The inverse Simpson and Shannon indices between the two sample origins within a given substrate were similar. According to the ACE index, frass and larvae reared on CD had a significantly lower species richness compared to the samples from FD-based substrates. For each of the substrates, a higher species ACE richness was observed in the frass compared to the larval intestine.

**Fig. 2:**
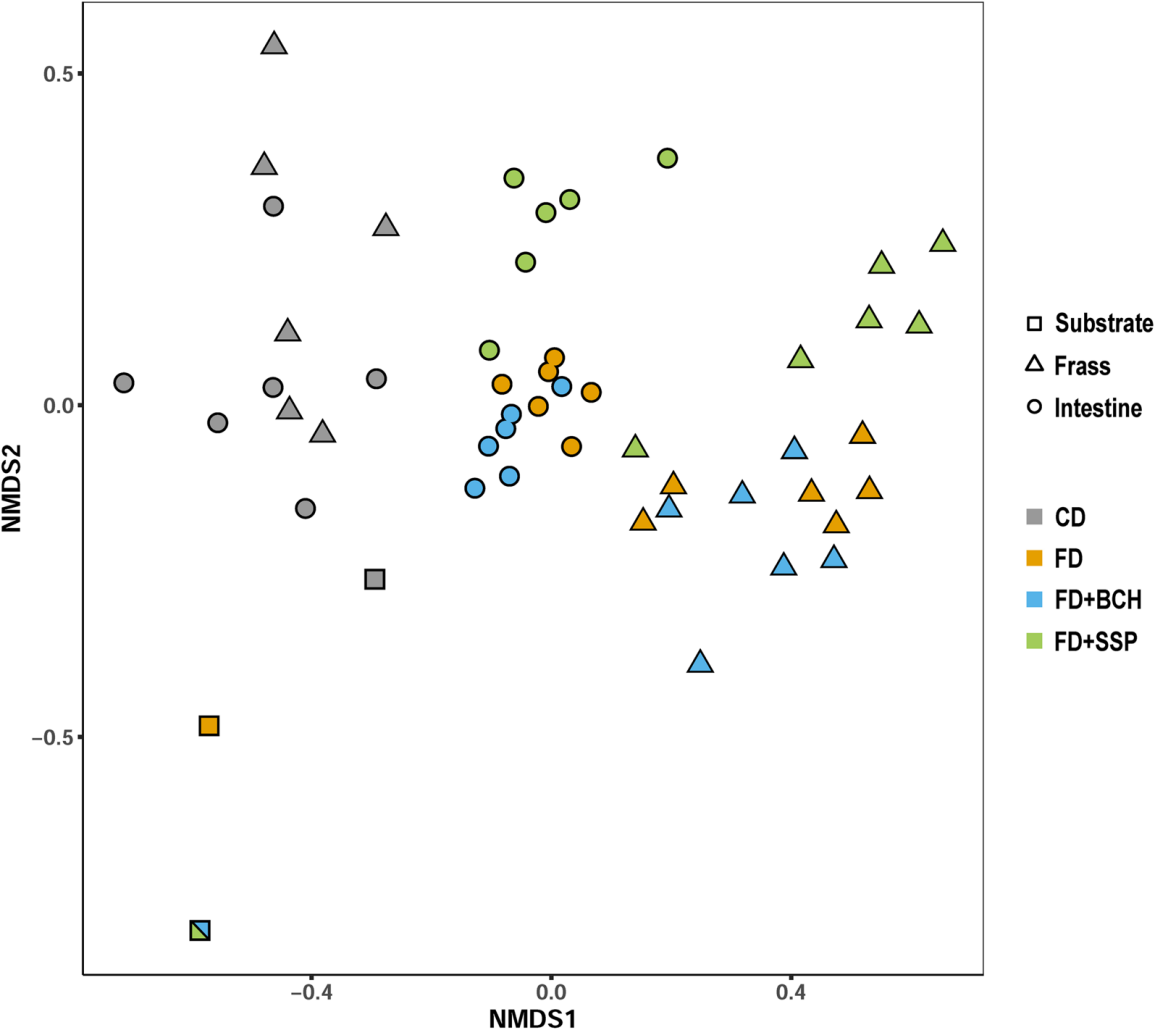
Microbial composition of substrate (square), frass (triangle), and black soldier fly larvae intestine (circle) samples represented by non-metric dimensional scaling (NMDS) ordination. The plot shows a sample of the substrates (rectangles) chicken feed diet (CD), fly diet (FD) and FD supplemented with biochar (FD + BCH) or single superphosphate (FD + SSP) recyclates after a 24-h pre-soaking procedure

The beta diversity analysis of the samples from BSFL intestine, frass, and substrates showed a significant differentiation between the samples derived from different substrates (*P* < 0.001) and the origin of the samples (*P* < 0.001) (Fig. 2). The microbial profiles of FD-based substrates (FD, FD + BCH, and FD + SSP) showed a clearer separation between frass and larvae samples as compared to the separation of the two sample origins within the CD substrate. For the FD-based diets, the NMDS plot indicated some divergence between FD + SSP and both FD and FD + BCH, whose clusters largely overlapped. Indeed, statistical analysis showed significant differences between the feeding substrates for both larval intestine and frass samples (*P*_adj._ < 0.05), except for the comparison of FD and FD + BCH (*P*_adj._ = 0.15; for frass: *P*_adj._ < 0.16; Additional file 1). The input substrates were spatially separated from the larval intestine and frass samples, with the exception of the CD substrate, which was slightly closer to the corresponding frass and larval gut samples. The taxaplot of the substrates fed showed that the top 14 microbial families overlapped in CD and FD (see Additional file 2).

**Fig. 3:**
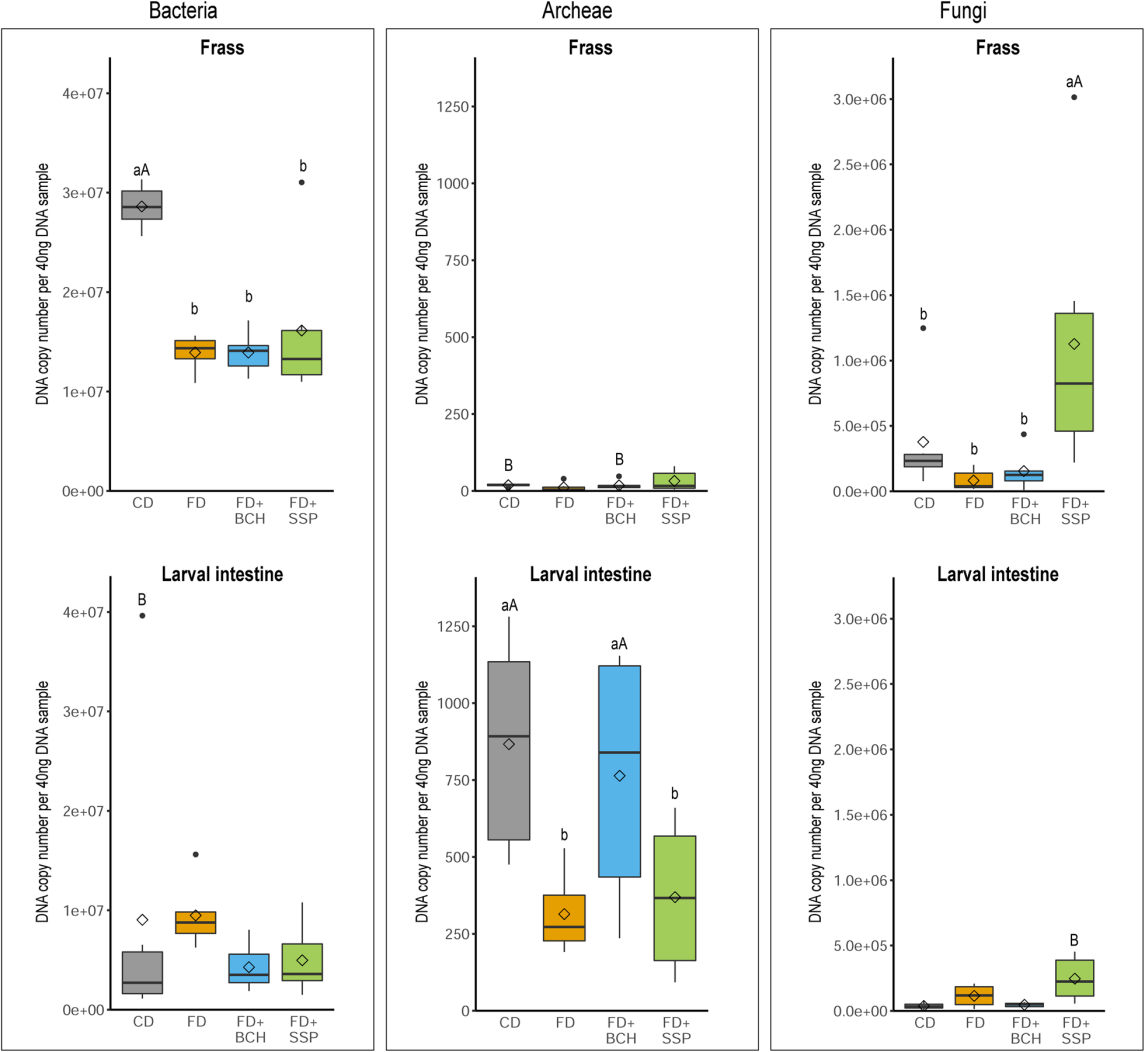
Abundance of bacteria, archaea, and fungi in frass and intestinal samples of black soldier fly larvae reared on chicken feed diet (CD), fly diet (FD) and FD supplemented with 4% biochar (FD + BCH) or with 3.6% single-superphosphate (FD + SSP) recyclates. The boxes give the 25th to 75th percentiles, the line the median, the diamond the mean, the whiskers the minimum and maximum values and the circles the outliers. ^abc^ and ^ABC^ indicate significant differences (*P* < 0.05) between feeding substrates within frass or larvae and for the same feeding substrate between frass and larvae, respectively

Based on the number of DNA copies of bacteria, archaea and fungi using quantitative PCR, an overview of the microbial composition in the frass and intestinal samples of BSFL was obtained. Bacteria represented the dominant fraction of the microbial community in the frass and larvae samples (Fig. 3). The frass samples revealed a higher abundance of bacteria in CD compared to all FD-based substrates, whereas similar abundances of bacteria were found in the larval intestinal tract for all substrates. Archaea was almost undetectable in the frass samples and were only present in very small quantities in the larval intestinal tract. The larval intestinal microbiota showed differences in the DNA copy number of archaea between CD and certain FD-based substrates. The addition of biochar (FD + BCH) resulted in a higher abundance of archaea compared to the basal FD substrate. The total amount of fungi in the frass and larvae samples was comparable. In frass, the addition of SSP to FD resulted in a higher abundance of fungi compared to the other FD-based diets. In the FD + SSP group, fungi were more abundant in the frass than in the larvae samples.

**Fig. 4:**
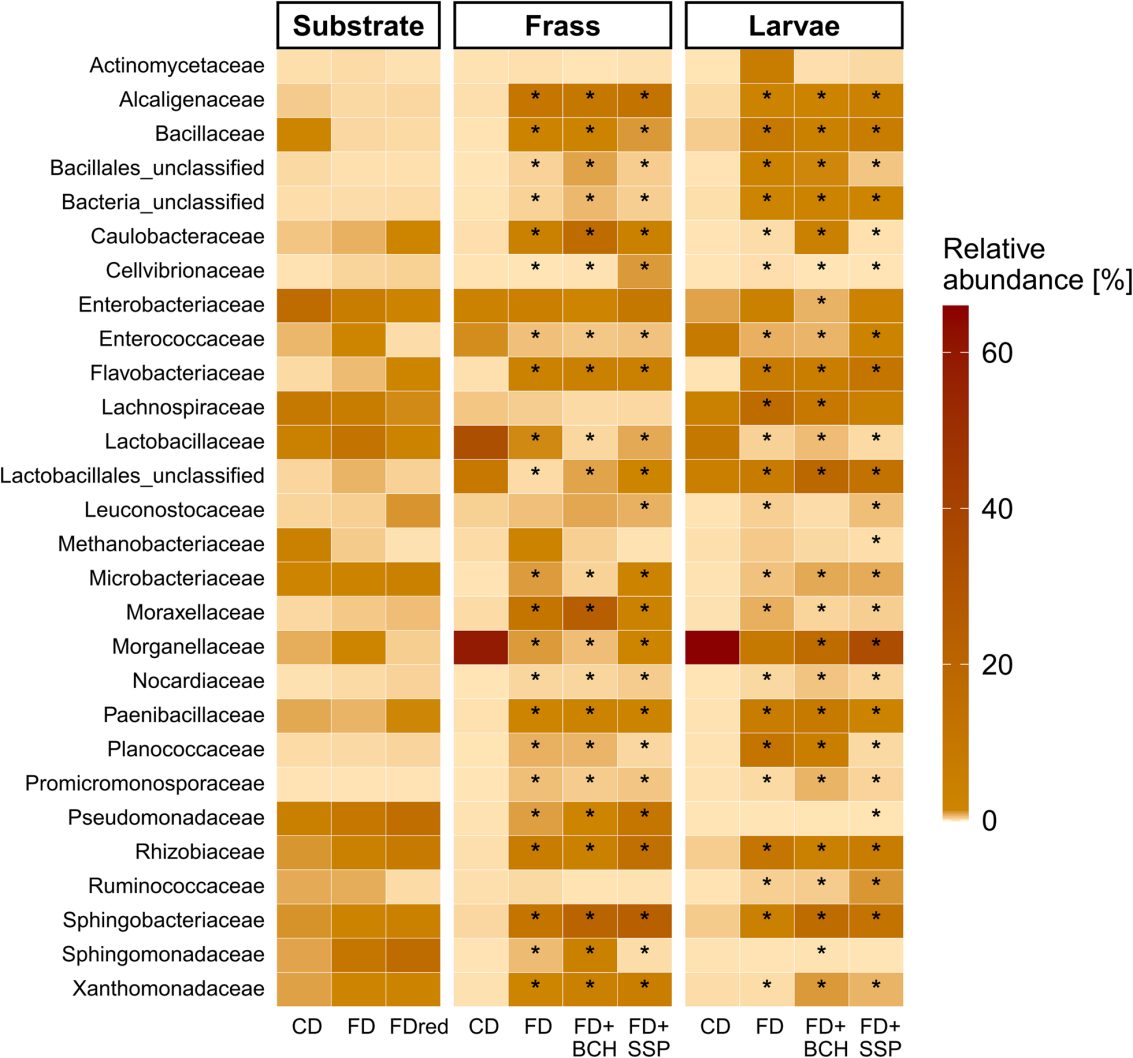
Mean relative abundance of microbial families in frass and intestinal samples from black soldier fly larvae reared on chicken feed diet (CD) compared to larvae reared on a modified fly diet (FDred), and a modified fly diet-based supplemented substrate (FD + BCH, FD + SSP). Families that passed the initial abundance filtering in the DESeq2 analysis were included. To illustrate the development of the microbial profiles, the microbial abundances of feeding substrates after a 24-h soaking period are shown (*n* = 1 sample per substrate)

^*^significantly different abundance of microbial taxa in FD-based groups compared to CD (adjusted *P*-value < 0.05).

Analysing the *16S* rRNA sequencing data for differential abundance with DESeq2 at the family level revealed a low microbial complexity of CD frass and BSFL intestine, which is characterised by only a few dominant families compared to the FD-based diets (Fig. 4, see Additional file 3). Although the microbial composition of the initial substrates was comparable, the microbial community in CD frass and larval intestine was dominated by *Morganellaceae* and families of *Lactobacillales*. In addition, *Enterobacteriaceae*, *Enterococcaceae*, and *Lachnospiraceae* were prevalent in the microbial communities of CD frass and larvae samples. Samples of FD-based diets revealed a microbial composition distributed across several families. They exhibited a similar pattern in frass and larvae, with minor shifts between the different mineral sources. Among the numerous abundant families, *Sphingobacteriaceae*, *Alcaligenaceae*, *Caulobacteraceae*, and *Rhizobiaceae* dominated in both the frass and larval gut samples. In the FD samples, microbes belonging to the *Flavobacteriaceae*, *Moraxellaceae*, and *Xanthomonadaceae* mainly contributed to the community in the frass, while *Lachnospiraceae*, *Morganellaceae*, and families of *Lactobacillales* were more represented in the intestine of larvae.

To further investigate the effects of biochar and SSP supplementation on the microbiota, comparative abundance analyses were performed at the genus level in the FD, FD + BCH and FD + SSP groups . The differences in microbiota composition between frass and intestine were also reflected at the genus level when comparing samples of larvae reared on FD-based substrates (Table 2). *Sphingobacterium* was the most abundant genus in the frass and accounted for an average of 25% of the microbial composition. Its abundance in the larval intestine was lower, but still considerable at around 9%. The genus *Morganella* (about 16%) was most abundant in the larval intestine, while it accounted for only 0.5% in frass. The supplementation of biochar to FD (FD + BCH) resulted in a lower abundance of *Escherichia-Shigella*, *Morganella*, and genera assigned to *Actinomycetaceae* and *Microbacteriaceae* in intestinal samples of the larvae compared to the other FD-based diets. In frass samples of FD + BCH, *Alcaligenes* and *Stenotrophomonas* were more abundant compared to FD and FD + SSP. Some specific differences were identified between the recyclates (FD + BCH vs. FD + SSP) in the relative abundance of *Brevundimonas* and genera classified as *Sphingomonadaceae* and *Actinomycetaceae* (Table 2). The inclusion of 3.6% SSP to the FD substrate (FD + SSP) significantly affected the abundance of *Ochrobactrum*, *Pseudomonas*, *Pusillimonas*, and *Stenotrophomonas* in frass and larval intestine compared to the other FD-based substrates. In larvae, a higher abundance of *Enterococcus* and a lower abundance of genera assigned to *Planococcaceae* as well as some changes in less abundant taxa of *Ammoniphilus*, *Methanobrevibacter*, and *Weissella* were observed in FD + SSP compared to FD and FD + BCH groups. Compared to the other substrates, a decrease in the abundance of *Acinetobacter* and an increase in the abundance of genera classified as *Enterobacteriaceae* was found in the frass of FD + SSP .

**Table 2:** Average relative abundance (%) of microbial genera in frass and intestinal samples from black soldier fly larvae reared on fly diet (FD) and FD supplemented with biochar (FD + BCH) or single superphosphate (FD + SSP)

	Frass^2^				Larval intestine^2^				*P*-value
Genus^1^	FD	FD + BCH	FD + SSP	SEM^3^	FD	FD + BCH	FD + SSP	SEM^3^	Sample origin
*Achromobacter*	1.57	2.26	2.01	0.63	0.06	0.11	0.24	0.08	0.001
*Acinetobacter*	11.28^a^	8.81^a^	3.39^b^	2.03	0.39	0.86	0.44	0.35	< 0.001
*Actinomyces*	0.28	0.13	0.46	0.16	1.94	3.18	2.42	1.25	0.003
uncl. *Actinomycetaceae*	0.034^ab^	0.001^b^	0.119^a^	0.04	0.98^a^	0.10^b^	2.21^a^	0.70	< 0.001
uncl. *Alcaligenaceae*	0.53	1.20	1.79	0.46	0.10	0.23	0.15	0.07	0.002
*Alcaligenes*	1.55^b^	5.07^a^	2.36^ab^	0.98	2.13	1.84	2.32	0.90	0.851
*Ammoniphilus*	0.10	0.06	0.09	0.02	0.65^a^	0.35^a^	0.06^b^	0.13	0.003
*Aquamicrobium*	1.27	1.17	0.72	0.37	0.88	1.30	0.65	0.34	0.832
uncl. *Bacillaceae*	1.92	1.66	0.98	0.44	5.30	7.05	3.57	1.29	0.003
uncl. *Bacillales*	0.64	0.43	0.53	0.15	2.69^a^	1.62^b^	0.73^b^	0.46	< 0.001
uncl. *Bacteria*	0.27	0.28	0.24	0.06	0.93	1.40	1.06	0.27	0.001
*Brevundimonas*	8.93^ab^	13.74^a^	2.62^b^	3.29	1.93	3.89	1.74	0.87	0.006
*Cellulosimicrobium*	0.43	0.43	0.44	0.11	0.42	0.69	0.39	0.12	0.841
uncl. *Enterobacteriaceae*	2.15^b^	0.91^b^	6.62^a^	0.62	0.94^b^	1.90^ab^	2.07^a^	0.47	0.016
*Enterococcus*	0.31	0.41	0.62	0.13	0.53^b^	1.02^b^	2.10^a^	0.44	0.053
*Escherichia-Shigella*	1.19	0.96	1.32	0.25	1.25^a^	0.26^b^	0.83^a^	0.50	0.832
*Flavobacterium*	5.46	3.74	5.36	0.95	4.48	2.69	5.37	1.62	0.406
uncl. *Lachnospiraceae*	0.24	0.13	0.12	0.05	6.33	7.48	3.88	1.48	< 0.001
uncl. *Lactobacillales*	0.42	0.62	1.20	0.18	5.88	13.21	10.54	1.80	< 0.001
*Methanobrevibacter*	0.63	0.33	0.26	0.23	0.20^a^	0.15^ab^	0.05^b^	0.04	0.363
uncl. *Microbacteriaceae*	0.98^a^	0.27^b^	1.01^a^	0.16	0.67^a^	0.46^b^	0.90^a^	0.16	0.230
*Morganella*	0.50	0.35	0.56	0.13	22.54^a^	5.97^b^	18.77^a^	6.28	< 0.001
*Ochrobactrum*	6.82^b^	3.69^b^	13.23^a^	0.97	3.99^b^	2.92^b^	7.06^a^	1.17	0.023
*Ornithinibacillus*	0.50	0.08	0.51	0.20	0.57^a^	0.06^b^	0.10^ab^	0.12	0.672
*Paenibacillus*	2.18	2.11	1.41	0.26	5.79	8.42	4.50	1.30	0.002
uncl. *Planococcaceae*	0.97^ab^	0.77^a^	0.3^b^	0.14	4.89^a^	5.13^a^	0.34^b^	0.68	< 0.001
*Providencia*	0.32	0.27	0.58	0.06	5.70	6.03	5.90	1.87	< 0.001
*Pseudomonas*	0.84^b^	2.33^ab^	8.03^a^	1.77	0.002^b^	0.031^b^	0.149^a^	0.041	< 0.001
*Pseudoxanthomonas*	5.21^ab^	1.77^a^	0.01^b^	0.98	0.026^a^	0.021^ab^	0.002^b^	0.010	< 0.001
*Pusillimonas*	5.55^a^	3.76^a^	0.19^b^	1.30	1.85^a^	1.03^a^	0.12^b^	0.46	0.152
*Rhodococcus*	0.30	0.22	0.25	0.06	0.32	0.55	0.24	0.08	0.786
uncl. *Ruminococcaceae*	0.08	0.06	0.05	0.04	0.60	0.89	0.18	0.19	0.003
*Sphingobacterium*	23.50	27.33	25.30	2.59	6.30	9.74	11.17	3.10	< 0.001
uncl. *Sphingomonadaceae*	0.89^ab^	2.87^a^	0.29^b^	0.43	0.006^ab^	0.063^a^	0.003^b^	0.030	< 0.001
*Stenotrophomonas*	1.27^b^	3.13^a^	7.85^a^	1.03	0.04^c^	0.32^b^	0.72^a^	0.12	< 0.001
*Weissella*	0.53	0.42	0.89	0.13	0.09^b^	0.07^b^	0.63^a^	0.09	< 0.001

^1^Genera with low abundance that failed the initial abundance filtering in the DESeq2 were excluded

^2^Six frass and six larval intestine samples were analysed for each substrate

^3^Pooled standard error of the mean for the relative abundance of microbial genera within frass and larvae

^abc^Different superscripts indicate significant differences in microbial abundance between the substrates within frass and larvae, respectively (adjusted *P*-value < 0.05)

**Fig. 5:**
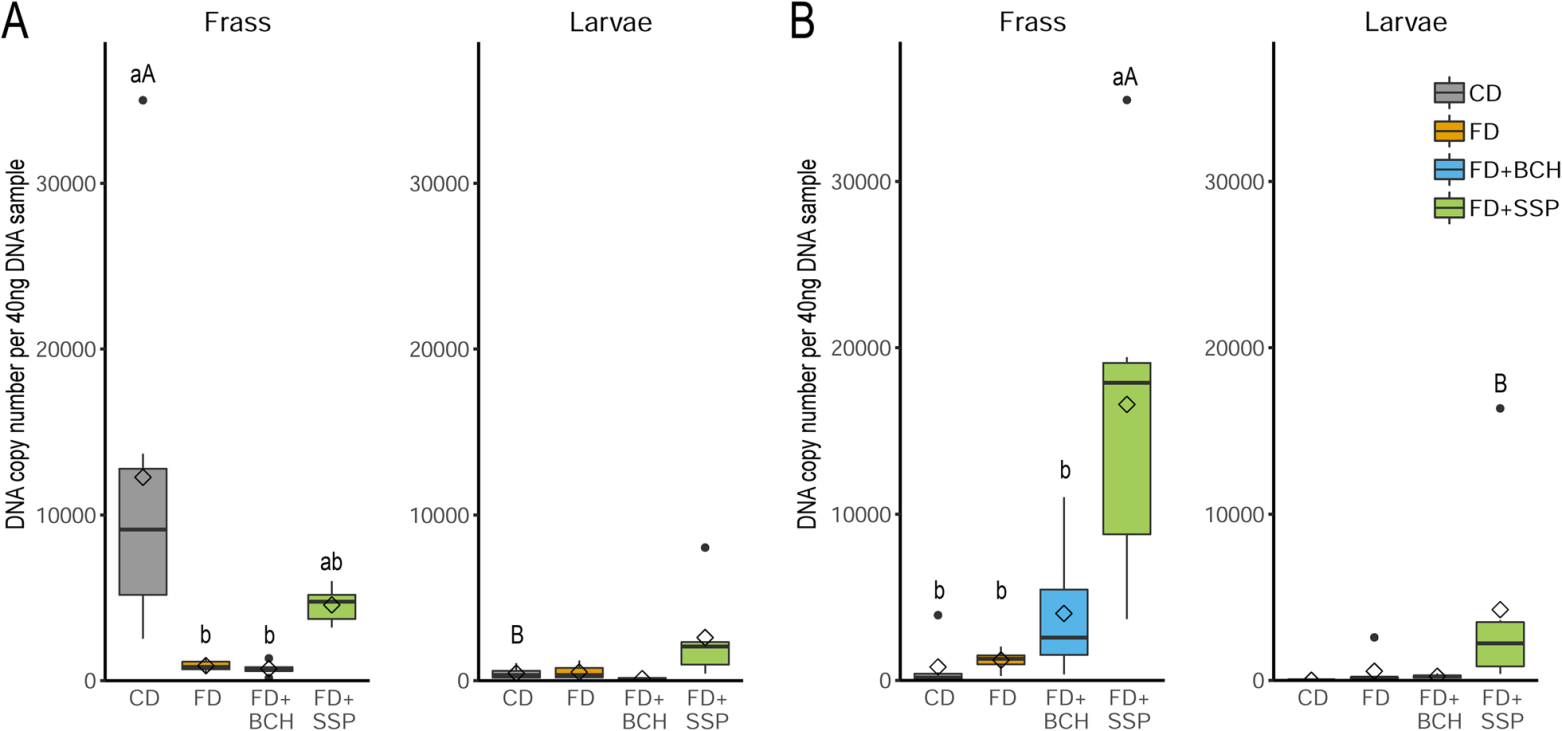
Abundance of *Pichia kudriavzevii* (**A**) and *Trichosporon asahii* (**B**) in frass and intestinal samples of black soldier fly larvae reared on chicken feed diet (CD), fly diet (FD) and FD supplemented with biochar (FD + BCH) or with single superphosphate (FD + SSP) recyclates. The boxes give the 25th to 75th percentiles, the line the median, the diamond the mean, the whiskers the minimum and maximum values and the circles the outliers. ^abc^ and ^ABC^ indicate significant differences (*P* < 0.05) between feeding substrates within frass or larvae intestine and for the same feeding substrate between frass and larvae intestine, respectively

On average, the two quantified fungal species were more abundant in the frass samples than in the larval intestine (Fig. 5). *Pichia kudriavzevii* was more abundant in CD frass samples than in frass of FD and FD + BCH, whereas *Trichosporon asahii* exhibited a higher abundance in frass samples of FD + SSP compared to the other substrates.

## Discussion

The two initial feeding substrates CD and FD differed substantially in their composition and nutrient availability but showed qualitatively similar microbiota profiles (Additional file 2). The CD had a considerably higher amount of fermentable starch than the FD, which probably promotes the initial development of the microbiota in the substrates as well as in the larval intestine and frass. In fact, the CD substrate generally led to increased bacterial proliferation , as indicated by the considerably higher bacterial abundance in CD frass compared to frass from FD-based diets. Because we used pelleted chicken feed, its thermal and pressure treatment might have affected nutrient accessibility. Therefore, the initial microbiota of the CD substrate might have been influenced due to changes in fermentability [22]. The FD substrate contained wheat bran and sugar beet pulp, which are rich fibre sources resulting in a stimulation of various microorganisms capable of fibre decomposition to increase nutrient availability. The nutrient composition of the substrates was also a major determinant of the development of the intestinal and frass microbiota of BSFL in other studies, although the effect varied between different substrates [23, 24]. The FD feeding substrate clearly clustered away from larval intestine and frass FD samples, while the CD-based samples clustered closer together. Consequently, the higher microbial diversity in the intestinal tract and larval diet with FD compared to CD substrates was reflected by alpha and beta diversity analyses. Dietary fibre has a fundamental impact on the composition of the gut microbiota, as demonstrated repeatedly in humans, with the degradation and availability of different types of fibre affecting the microbial diversity [25]. This is also true for the microbiota of insects that are fed on fibre-rich substrates such as bamboo, which leads to a shift towards cellulolytic bacterial communities [26]. The microbial profiles of FD-based samples compared to CD showed an enrichment of several families previously described to include cellulolytic species, such as *Bacillaceae*, *Enterobacteriaceae*, *Microbacteriaceae*, *Paenibacillaceae*, and *Promicromonosporaceae* [27]. In contrast, in larval intestine and frass of the CD group families of *Lactobacillales* and *Morganellaceae* were dominant, which is in accordance with other BSF studies using chicken feed [18]. The larger amount of readily available nutrients in the CD substrate favoured faster growing microorganisms that prevail. The fungus *Pichia kudriavzevii* was more abundant in CD than in FD-based substrates, but its abundance was modulated between the larval intestine and frass. *Pichia kudriavzevii* has been consistently detected to be one of the most common fungal species in BSF, with reported antimicrobial functions and effects on substrate degradation [21, 28]. Frass from BSFL has a valuable nutritional profile for plants, making it a useful agricultural fertiliser that improves growth, yield, and nutritional quality of various crops [29]. For example, FD-based frass samples showed enrichment with *Pseudomonadaceae*, which are known to have a positive effect on plant growth, but also enrichment of *Xanthomonadaceae*, some species of which have been described as causing plant diseases [29, 30]. Overall, studies on the effect of different rearing substrates of BSFL reported a high adaptability of the larvae and a modulation of the substrate to generate optimal conditions for biodegradation and larval growth [31].

The supplementation of the FD substrate with sewage sludge-derived products did not result in differences in larval body mass, although there was a trend towards lower final body mass in the FD + SSP group. As previously reported, the addition of both BCH and SSP to FD significantly increased the Ca content of BSFL, while the addition of SSP only increased the P level [7].

An additional aspect to consider when using recycled sewage sludge in the rearing of BSFL is the risk of heavy metal accumulation. We reported earlier that larvae fed with FD + BCH in this study showed increased levels of iron, cadmium and lead compared to the basal FD substrate, whereas larvae fed on FD + SSP had increased levels of arsenic and lead compared to FD [7]. In general, heavy metals in certain concentrations are toxic to microorganisms and can alter the microbial profiles of substrates [32]. For BSFL, high concentrations of cadmium and copper in the substrate may affect the intestinal microbial profiles but without impairing larval growth [33]. However, even the lowest tested doses of 100 mg/kg feed for copper and 10 mg/kg feed for cadmium led to a much higher accumulation in BSFL than in the BSFL of the current study [7]. Accordingly, the effects of heavy metal levels in the feed substrate, especially heavy metals other than copper and cadmium, on the BSFL microbial community cannot be clearly deduced in our study and require a specific experimental design with defined heavy metal levels.

For FD + BCH, the microbiota profiles were only slightly different from the basal FD substrate. Biochar is a highly porous material with an increased surface area that can improve the water storage capacity, the pH value and nutrient exchange [34, 35]. The release potential of minerals, e.g. of P from biochar is still under dispute, but appears to be influenced at least by the source material of biochar production and the processing properties [4, 36]. In addition, biochar can influence the microbial community and can limit the spread of pathogens in substrates [37]. It has been described that the addition of biochar can decrease the burden of the protozoon parasite *Cryptosporidium parvum* [38] and reduce the mycotoxin content, e.g. intestinal *Fusarium* toxins are adsorbed to a high degree by biochar [39]. Considering the properties of biochar and the results of the current study, the addition of biochar to the FD substrate might contribute to control the substrate conditions, thereby limiting potential pathogens such as *Escherichia-Shigella*, but without impacting the P supply to the BSFL.

The addition of SSP to the FD substrate has a more pronounced effect on the microbiota compared to the addition of biochar. From plant and soil research, several microbes, so-called phosphate-solubilising microorganisms, have been identified, which are known for their ability to solubilise inorganic P from insoluble compounds [40]. Similar contributions of microorganisms to increasing the available P concentration seem to apply to BSFL [23]. A typical representative of phosphate-solubilizing bacteria is *Pseudomonas* [23, 40], which we found significantly more abundant in the frass and larval intestine of FD + SSP compared to the other substrates. Other described phosphate-solubilising taxa with a similar prevalence in the FD + SSP substrate were *Ochrobactrum* [41], *Stenotrophomonas* [42], and genera of *Enterobacteriaceae* [43]. Furthermore, it has been reported that the occurrence of fungi appears to be related to various mineral sources and that fungi can effectively promote the dissolution of minerals [44]. Fungi in general and *Pichia kudriavzevii* and *Trichosporon asahii* in particular exhibited an enrichment in the FD + SSP substrate. The SSP is derived from the Ash2Phos process, which has high recovery rates for P of up to 95% and yields the P- and Ca-rich product Ca_5_(PO_4_)_3_OH [45]. However, an earlier study on monogastric farm animal species showed that the addition of precipitated Ca phosphate to the diets resulted in a lower digestibility compared to conventional monocalcium phosphate [46]. Thus, the aforementioned changes in the microbial community with the proliferation of phosphate-solubilising microbes due to SSP supplementation could therefore lead to increased P bioavailability in the larvae.

## Conclusions

The results of this study and the available literature consistently demonstrate that BSFL exhibit an enormous capacity to adapt to changing environmental conditions, i.e. feeding substrates. Microbial diversity is a prerequisite for an efficient larval rearing system and the result of the interaction between the microbiota of the larvae and the substrate.

The nutrient-rich and easily fermentable CD substrate enhanced larval growth performance but reduced microbial diversity in the frass and larval intestine samples. In contrast, high-fibre FD diets offer the advantage that they fully exploit the potential of the holobiont in substrate conversion for sustainable production. The addition of biochar to the FD substrate (FD + BCH) appeared to promote substrate conditions for microbial development and for larval Ca supply but did not serve as an additional P source for BSFL. The supplementation with single superphosphate (FD + SSP) promotes *Pseudomonas* and fungi abundance, which were previously ascribed to have phosphate-solubilising properties, and might contribute to increase the P availability from the substrate. This highlights some specific mechanisms by which BSFL and its microbiota can interact in the biological transformation of substrates, utilising the nutrient profile of sewage sludge-derived products. Whether this occurs as an adaptation of the microorganisms to the available nutrients in the substrate or is actively influenced by the larvae would be interesting to investigate further and could possibly be tested by targeted microbial inoculation of substrates.

## Materials and methods

### Growth conditions and sample collection

The BSF population used in the study originated from the Hermetia Baruth GmbH company (Baruth, Germany). The hatching of the larvae, housing conditions, and the feeding trial were previously described in detail [7]. In brief, the feeding trial was conducted in two runs starting with 5-day-old larvae after an initial rearing on chicken starter feed. Larvae were sieved to separate them from the chicken feed starter substrate and a larval mass representing approximately 8,000 individuals was placed in plastic boxes (40 × 60 × 22 cm) with the respective experimental feeding substrate. The ingredients and analysed chemical composition of the FD-based substrates are given in Seyedalmoosavi et al*.* [7], while the analysed composition of the CD is shown in Additional file 4. The substrates containing recyclates were formulated based on a modified Gainesville FD at the expense of wheat bran and corn meal (FDred) with either 4% BCH or 3.6% SSP normalised to dry matter (DM). The BCH was prepared from dried sewage sludge by carbonisation at 500–700 °C in the absence of oxygen (PYREG process; PYREG GmbH, Dörth, Germany). The SSP was produced from ash resulting from the combustion of sewage sludge and a further acid treatment to reduce the concentration of heavy metals (Ash2Phos process; EasyMining Sweden AB). Compared to BCH, the SSP recyclate had a higher content of minerals but a lower content of heavy metals [7]. In each of the two runs, three rearing containers per substrate were used, except for FD, where 2 and 4 rearing containers were used per run. Thus, a total of 6 replicates were performed for each feeding substrate. The feeding substrates for BSFL were produced by mixing with water in a ratio of 3 (storage moisture) to 7 and pre-soaked for 24 h. The respective substrates were fed on days 5, 9 and 13 after hatching in quantities per box of 2,500 g, 4,000 g and 3,500 g, respectively. The larvae were sampled at the beginning of the pre-pupae stage (i.e. approximately 10% in pre-pupal stage) 18 to 20 days after hatching. For this purpose, the larvae were separated from the substrate by sieving, then rinsed under tap water and dabbed with a soft paper towel. Larvae specimens were devitalised in liquid nitrogen and stored at − 80 °C until further processing. Corresponding frass samples were taken from each replicate box. In addition, samples of the pre-soaked substrates were taken and stored at -20 °C. At least 100 larvae per box were counted and weighed together to determine the larval body mass. The dry mass was determined from 2 g of a separate sample using a Sartorius MA 35 moisture analyser (Sartorius, Göttingen, Germany). Additional frozen larvae from each rearing container were used for the chemical analysis of minerals (Ca, P, Mg, Na, K, Fe, Zn, Cu, Cd, PB, and Mn) as described [7]. Phenotypic data (body mass, dry mass, nutrient, mineral, and heavy metal contents of BSFL) were analysed with a one-way analysis of variance (ANOVA; feeding substrates: CD, FD, FD + BCH, FD + SSP) and group comparisons were carried out with the Tukey HSD post-hoc test in R (version 4.2.0). This analysis differed from Seyedalmoosavi et al*.* [7] by the additional consideration of the CD group.

### Larvae preparation and DNA extraction

Prior to processing, the intact frozen larvae were carefully rinsed with distilled water. The isolation of the larval gastrointestinal tract (GIT) was performed under the microscope in a Petri dish with ice-cold PBS. The larvae body was carefully opened with scissors, fixed with needles and the intestinal tract was extracted with forceps. The equipment was disinfected or changed between dissections. At least three larvae were prepared and combined for each of the six boxes per substrate. DNA extractions from pooled GIT (n = 6 / substrate), frass (n = 6 / substrate) and substrate (n = 1 / substrate) samples was performed using the PowerLyzer PowerSoil DNA Isolation Kit according to the manufacturer’s instructions (QIAGEN, Hilden, Germany). To improve sample disintegration, additional incubation steps were performed for 10 min at 70 °C and 10 min at 95 °C, as well as a mechanical breakdown using a Precellys 24 homogeniser (PEQLab Biotechnology GmbH, Darmstadt, Germany) and beads provided in the PowerLyzer PowerSoil DNA Isolation Kit.

### 16S rRNA gene sequencing of microbiota

For targeted sequencing of the hypervariable region 4 of the *16S* rRNA gene, specific amplicons were prepared with primers 515′F and 806R [47, 48]. The PCR was performed in duplicate using GoTaq G2 Hot Start Polymerase (Promega, Mannheim, Germany) under standard conditions with 35 cycles and an annealing temperature of 50 °C. Amplification products were purified with the SequalPrep Normalization Plate (Thermo Fisher Scientific, Darmstadt, Germany) and sequenced on a HiSeq 2500 instrument (Illumina, San Diego, CA, USA) at a length of 2 × 250 bp. This resulted in an average of 627,262 ± 286,578 pair-end reads per sample. The sequences were pre-processed and prepared according to the mothur pipeline (version 1.47.0) with Silva as the reference database (release v138.V4; https://www.arb-silva.de/) [49]. Sequences with any ambiguous bases, with homopolymers longer than six bp, with a length of more than 275 bp and with chimeras were removed during processing. Using mothur, operational taxonomic units (OTU) were derived from the sequences, clustered based on a sequence identity of ≥ 97% and subsequently annotated at genus level.

### Quantification of specific taxa

Primer pairs specific for bacteria (Eub338 and Eub518 [50]), archaea (Arch-967F and Arch-1060R [51]), and fungi (FR1 and FF390 [52]) were used to determine their abundance in each sample (see Additional file 5). To quantify specific fungal species, primers were designed for *Pichia kudriavzevii* (Pichia_f1 and Pichia_r1; XM_029467014.1), one of the most prevalent representatives of *Ascomycota* in BSFL [17], and *Trichosporon asahii* (Tricho_A_F1 and Tricho_A_R1 [53]), as one of the dominant *Trichosporonaceae* [54]. Real-time PCR analysis was performed in duplicates on a LightCycler 480 instrument with the LightCycler 480 SYBR Green I Master (Roche, Mannheim, Germany) and an input of 40 ng DNA per sample. The DNA concentrations of all final sample dilutions were measured with the Nanodrop 2000 instrument (Thermo Fisher Scientific, Dreieich, Germany). The amplification comprised an initial denaturation at 95 °C for 5 min, followed by 45 cycles with 10 s at 95 °C, 15 s amplification, and 25 s extension at 72 °C. Amplified products were subjected to melting curve analyses to check the specificity of amplification. For all assays, the DNA copy number was determined from a standard curve of serial dilutions of a corresponding PCR standard (10^7^–10^2^ copies). The sample-specific DNA concentrations of final sample dilutions were used for factorial normalisation of abundance values. The quantification of specific taxa was statistically analysed using a linear model considering the interaction between sample origin (i.e. larvae intestine, frass) and substrate as well as the box effect. Differences with a *P*-value < 0.05 were declared significant.

### Analysis of 16S rRNA sequencing data

Microbial alpha diversity was assessed at the OTU level using the Shannon index, the Simpson index, and the abundance-based coverage estimator (ACE) included in the R package phyloseq [51]. Differences in diversity indices between groups were examined with the Kruskal–Wallis test. The Wilcoxon exact rank sum test was used for pairwise comparisons. Beta diversity was visualised in a non-metric multidimensional scaling (NMDS) plot based on a Bray–Curtis dissimilarity matrix. Using this distance matrix, a permutational multivariate analysis of variance was performed on the factors feed substrate and sample origin (larvae intestine, frass, diet) with the vegan package in R (https://vegandevs.github.io/vegan/). Pairwise comparisons of microbial communities across all contrasts were performed using the pairwiseAdonis package (https://github.com/pmartinezarbizu/pairwiseAdonis) in R, identifying significant differences with a Benjamini–Hochberg adjusted *P*-value < 0.05. Taxaplots were created in R for the samples of the pre-swollen substrates of CD, FD, and the FD used as the basis for the recyclate supplementation.

The analyses on differential abundance were performed with the negative binomial framework implemented in the DESeq2 R package [56]. The initial filtering steps included a subsampling to the samples with the lowest number of OTU counts (20,841) and a pre-filtering of low abundance taxa to include only taxa with more than 100 counts in at least six samples. The read counts were normalized in DESeq2 according to the sequencing depth. The statistical model for the analysis on family level included the interaction of substrates (CD, FD, FD + BCH, and FD + SSP) and sample origin (larvae intestine and frass). The analysis at genus level focused on FD-based substrates (FD, FD + BCH, and FD + SSP) and considered different substrates, sample origin and their interaction as fixed effects. CD was excluded from the analysis at genus level, as the high degree of differentiation between CD and FD-based microbiota profiles was already reflected at family level. *P*-values were corrected using the Benjamini–Hochberg multiple testing approach, and adjusted *P*-values < 0.05 were considered significant.

## Supplementary Information

Additional file 1.

Additional file 2.

Additional file 3.

Additional file 4.

Additional file 5.

## Data Availability

Data are available from the NCBI database via the accession number PRJNA1167463: https://dataview.ncbi.nlm.nih.gov/object/PRJNA1167463?reviewer = as8n3hrsho0inmr143cjmvio05.
