# Supplementary figures and images for: Microbial profiling of black soldier fly larvae reared on substrates supplemented with different mineral sources originating from phosphorus recycling technologies
Source: Anim Microbiome. 2025 Feb 11;7:14. doi: 10.1186/s42523-025-00380-5 (PMC11812260; doi:10.1186/s42523-025-00380-5)

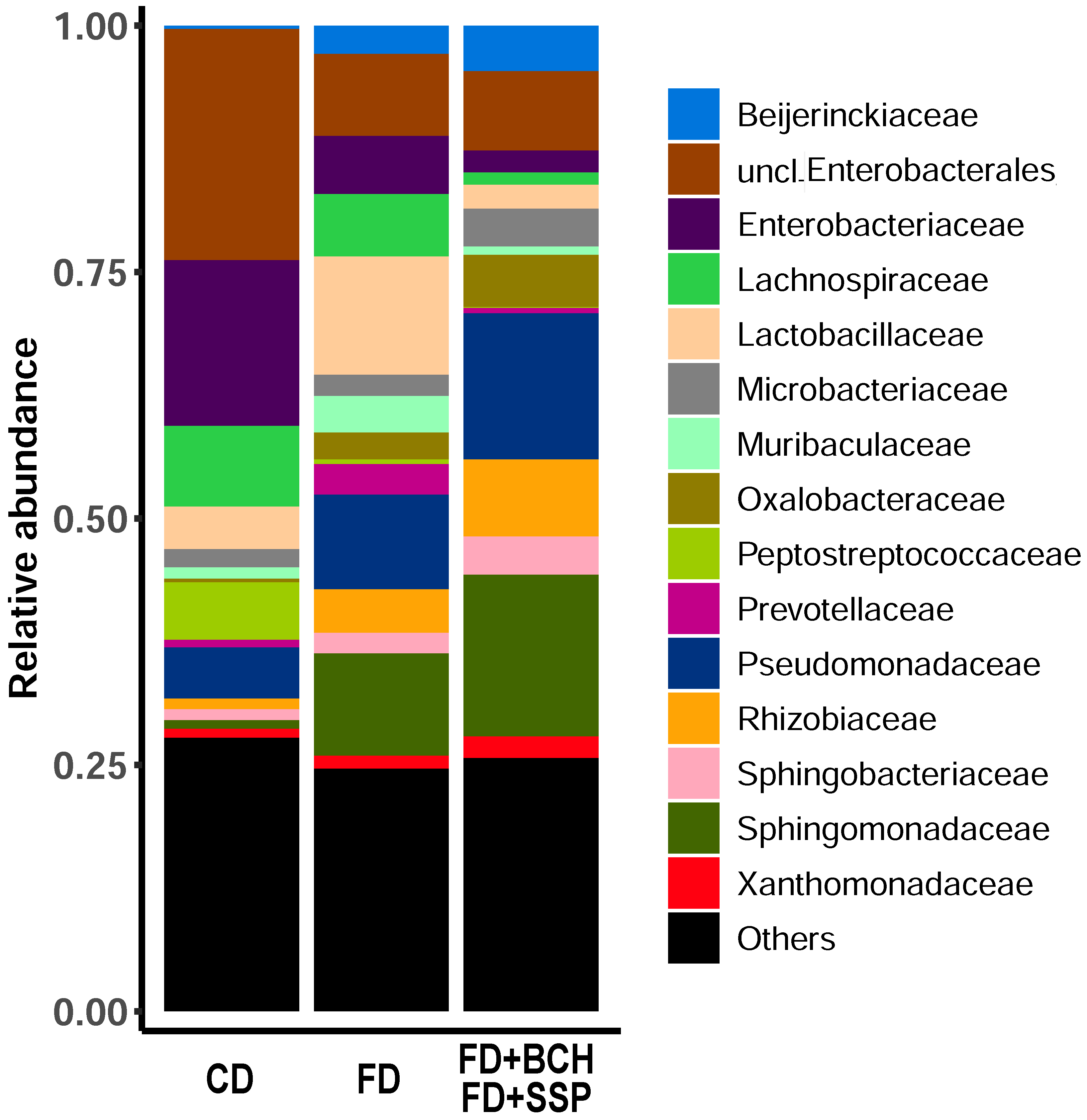

Supplement: Supplementary file 2 — Additional file 2. [file 42523_2025_380_MOESM2_ESM.tif]
