# Supplementary material for: Microbial profiling of black soldier fly larvae reared on substrates supplemented with different mineral sources originating from phosphorus recycling technologies
Source: Anim Microbiome. 2025 Feb 11;7:14. doi: 10.1186/s42523-025-00380-5 (PMC11812260; doi:10.1186/s42523-025-00380-5)
Supplement: Supplementary file 4 — Additional file 4. [file 42523_2025_380_MOESM4_ESM.docx]

**Additional file 4**. Ingredients and chemical composition of the chicken feed substrate fed to black soldier fly (*Hermetia illucens*) larvae.

| Items | **Chicken feed substrate^1,2^** |
| --- | --- |
|  |  |
| ***Ingredients, % FM*** |  |
| Wheat | 58.8 |
| Soybean meal HP | 24.3 |
| Corn grain | 10.0 |
| Fine Lime (Vitacarb 15) | 2.0 |
| MCP monocalcium phosphate | 1.25 |
| Premix J 408 Salvana^3^ | 1.1 |
| Soybean oil | 1.0 |
| NaCl | 0.35 |
| Methionine hydroxy analog | 0.20 |
| Lysine sulfate BB | 0.05 |
| ***Nutrient composition, g/kg DM*** |  |
| Dry matter, g/kg FM | 880 |
| Crude protein | 209 |
| Crude fat | 19 |
| Crude fibre | 46 |
| Crude ash | 58 |
| Starch | 460 |
| Total sugar (calculated as saccharose) | 46 |
| aNDFom | 167 |
| ADFom | 64 |
| ME, MJ/kg DM | 12.5 |
| GE, MJ/kg DM**^4^** | 17.2 |
| ***Macrominerals, g/kg*** ***DM*** |  |
| Ca | 9.0 |
| P | 7.9 |
| Mg | 2.1 |
| Na | 1.6 |
| K | 10.3 |
| ***Heavy metals, mg/kg*** ***DM*** |  |
| Mn | 136 |
| Fe | 239 |
| Zn | 105.5 |
| Cu | 12.10 |
| As | 0.11 |
| Cd | 0.06 |
| Pb | 0.11 |
| Hg | 0.23 |

**^1^**Manufactured by Trede und von Pein GmbH, Itzehoe, Germany

**^2^**The feeding substrate given to the larvae is comprised of a mixture of 30% dry feed (storage humidity) and 70% water.

^3^Amount of vitamin and minerals provided through premix per kg of feed are as following; Vit. A 10000 IU, Vit. D3 2000 IU, Vit. E 20 mg, Vit. K3 3 mg, Vit. B1 1 mg, Vit. B2 6 mg, Vit. B6 3 mg, Vit. B12 30 mcg, Niacin 30 mg, Pantothenic acid 10.8 mg, Folic acid 0.4 mg, Biotin 24 µg, Cholin 300 mg, Fe 55 mg, Cu 18 mg, Zn 80 mg, Mn 93 mg, I 0.66 mg, Se 0.34 mg, Co 0.05 mg, Phytase 250 FTU.

^4^ GE (gross energy) was calculated from ME (Metabolizable energy) by multiplying with the conversion factor of 1.379 (NRC, 1994).

**Abbreviations:** FM: Fresh matter; DM: Dry matter; aNDFom: Neutral detergent fibre after amylase treatment (organic matter); ADFom: Acid detergent fibre (organic matter).
