# Supplementary material for: Microbial profiling of black soldier fly larvae reared on substrates supplemented with different mineral sources originating from phosphorus recycling technologies
Source: Anim Microbiome. 2025 Feb 11;7:14. doi: 10.1186/s42523-025-00380-5 (PMC11812260; doi:10.1186/s42523-025-00380-5)
Supplement: Supplementary file 5 — Additional file 5. [file 42523_2025_380_MOESM5_ESM.docx]

**Additional file 5.** Sequence information of taxa-specific primers.

| **Primer** | **Sequence** | **Annealing temp. (°C)** | **Amplicon length (bp)** |
| --- | --- | --- | --- |
| Arch-967F | AATTGGCGGGGGAGCAC | 58.0 | 140 |
| Arch-1060R | GGCCATGCACCWCCTCTC |  |  |
| Eub338 | ACTCCTACGGGAGGCAGCAG | 58.0 | 200 |
| Eub518 | ATTACCGCGGCTGCTGG |  |  |
| FR1 | AICCATTCAATCGGTAIT | 50.0 | 280-390 |
| FF390 | CGATAACGAACGAGACCT |  |  |
| Pichia_f1 | GGCGTTGTCCATCCAATG | 58.0 | 133 |
| Pichia_r1 | TCGCAGAATGTGTAGGTGATG |  |  |
| Tricho_A_F1 | GCGACCTCAGCATCTTAATCA | 57.0 | 251 |
| Tricho_A_R1 | CTCTGAGGCCTTGCTCCTGT |  |  |
